# Aberrant RNA identification reveals triggers of transgene silencing

**DOI:** 10.1093/plcell/koaf274

**Published:** 2025-11-13

**Authors:** Andrew C Willoughby

**Affiliations:** Assistant Features Editor, The Plant Cell, American Society of Plant Biologists; Department of Biology, Duke University, Durham, NC 27708, USA; Salk Institute for Biological Studies, La Jolla, CA 92037, USA

Transgene silencing remains a major challenge in plant biotechnology, undermining the reliability of genetically engineered traits designed to improve crop productivity and resilience and confounding plant biology research into gene function (Rajeevkumar et al. 2015). A new study by **Marianne C. Kramer and colleagues (Kramer et al. 2025)** introduces 2 technologies that together illuminate a molecular trigger responsible for the initiation of transgene silencing in plants. This work provides unprecedented mechanistic insight into how certain plant transgenes are marked for silencing, revealing that specific coding sequence features can initiate the process independent of promoter activity or species context. By deploying advanced sequencing and analytical methods, the authors offer a roadmap for the rational design of transgenes that are resistant to silencing, with implications for crop improvement and trait durability.

The authors developed a visual transgene silencing system using a constitutively expressed RUBY reporter construct, which encodes 3 enzymes that produce the red pigment betacyanin throughout the plant (He et al. 2020). This system enables precise identification of active (red) or silenced (green) states of transgene expression, as well as transitional states, even in a single organ, offering a visual correlate to underlying molecular events (Fig.). Alongside this, the authors created the “All-in-One” RNA sequencing (AIO-seq) method, combining target capture enrichment and long-read sequencing to identify all transcripts arising from select loci, both polyadenylated and nonpolyadenylated. This allows for both qualitative and quantitative insights into transcript structure, abundance, and processing (Kramer et al. 2025).

**Figure:**
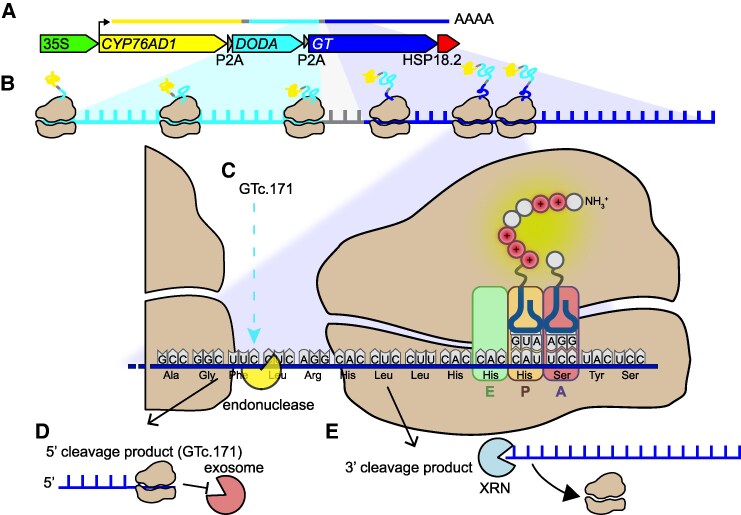
Ribosome stall leading to silencing. Polyadenylated RUBY transcript **(A)** is translated by ribosomes **(B)**. Inherent to the RUBY transcript is an HHH motif that causes ribosomal stalling at GTc.171 **(C)**, leading to cleavage by unknown endonucleases. The 5′ fragment is protected from 3′ degradation by an upstream ribosome, allowing it to be robustly detected by AIO-seq **(D)**, while the 3′ fragment is degraded by 5′->3′ XRN exonucleases **(E)**. In red tissues, although some siRNAs can be detected, translation proceeds and generates a red phenotype. In green tissues, the accumulation of GTc.171 transcripts leads to an increase in the generation of siRNAs, causing RNAi and cleavage of other full-length polyadenylated RUBY transcripts and subsequent feed-forward siRNA generation and silencing. Adapted from Kramer et al. (2025), Figure 9.

By integrating RUBY phenotyping and AIO-seq in both Arabidopsis (*Arabidopsis thaliana*) and lettuce (*Latuca sativa*), Kramer et al. show that an aberrant truncated RNA (GTc.171) arising from cleavage within the RUBY transcript strongly correlates with the onset of silencing (Fig.). This aberrant RNA accumulates prior to the rise of small interfering RNAs (siRNAs) and post-transcriptional gene silencing in transitional tissue, implicating it as an upstream trigger. The authors demonstrate that the cleavage at GTc.171 results from ribosome stalling at a rare triplet histidine (HHH) sequence encoded in the transgene. Ribosome stalling within the RUBY transcript induces No-Go RNA Decay, a translation-coupled quality control pathway. No-Go RNA Decay initiates endonucleolytic cleavage of the mRNA, generating the GTc.171 aberrant RNA, and when this accumulates to high levels, they are subsequently fed into siRNA-generating pathways. Mutational experiments altering the HHH motif (either through amino acid substitution or frameshift) significantly reduced aberrant RNA production and cleavage events, confirming the role of this motif in triggering silencing. Therefore, silencing of the RUBY construct in this system depends on the nature of the coding sequence rather than other features of the transgene, such as the promoter used or copy number, which are also known to influence transgene silencing (Schubert et al. 2004; Kramer et al. 2025). The findings suggest that in some cases, transgene silencing in plants can be mitigated by the careful design of coding sequences to avoid repeated charged amino acid motifs that promote ribosome stalling. This could be an important consideration in engineering improved crop species used in agriculture, pharmaceuticals, and biomedicine.

## Recent related articles in *The Plant Cell*

- Ohnishi and Kawashima (2023) reported unexpected transgene silencing in *Arabidopsis thaliana* sperm cells.
- Li et al. (2025) reported on the impairment of RNA silencing by ALTERED MERISTEM PROGRAM1, which represses the biogenesis of small interfering RNAs derived from a subset of inverted repeats in Arabidopsis.
- Vaucheret and Voinnet (2024) reviewed the biogenesis of plant siRNAs and the variety of their known or presumed functions.
